# Comparative evaluation of a basic life support educational model in non-health university students: the “*Lives to Give Life*” project and its social and health impact

**DOI:** 10.3389/fmed.2026.1765154

**Published:** 2026-03-10

**Authors:** Pedro Fernández Florido, Francisco Manuel Parrilla Ruiz, Gerardo Gómez Moreno, Ana Carrasco Cáliz, José Miguel Pérez Villares, Antonio Cárdenas Cruz

**Affiliations:** 1 Intensive Care Service, Hospital Universitario Virgen Macarena, Sevilla, Spain; 2 CTS-609 Research Group, Granada, Spain; 3 Emergency Service, Hospital Universitario Clínico San Cecilio, Granada, Spain; 4 Department of Medicine, University of Granada, Granada, Spain; 5 Research Group TEC 23, Instituto de Investigación Biosanitaria de Granada (IBS Granada), Granada, Spain; 6 Department of Stomatology, Faculty of Dentistry, Colegio Máximo de Cartuja s/n, University of Granada, Granada, Spain; 7 CTS-654 Research Group, Granada, Spain; 8 Intensive Care Service, Hospital Universitario Virgen de las Nieves, Granada, Spain; 9 Faculty of Medicine, Universidad de Granada, Granada, Spain; 10 Research Group, Plan Andaluz de Investigación, Desarrollo e Innovación (PAIDI), Granada, Spain

**Keywords:** basic life support, cardiac arrest, health promotion, nonhealth degrees, public health impact, resuscitation, teacher training, university education

## Abstract

**Introduction:**

Out-of-hospital cardiac arrest is a global public health problem with high rates of preventable mortality. Teaching basic life support to the general population is a key strategy for improving survival, although its implementation is often restricted to the healthcare setting. This study evaluates the potential benefits and social impact of an innovative model of basic life support teaching and learning applied to university professors in non-health-related degrees, within the *Lives to Give Life* project, developed at the University of Granada (Spain).

**Methods:**

A cross-sectional study was designed between two groups of students: one consisting of healthcare professionals and the other of non-health-related university professors previously trained in basic life support. Theoretical knowledge and practical skills were analyzed using structured objective tests, assessing the correct execution of essential maneuvers, such as recognition of cardiac arrest, activation of the emergency system, cardiopulmonary resuscitation, and use of the automated external defibrillator. Descriptive and inferential analyses were performed, considering *p* < 0.05 as the level of significance.

**Results:**

Both groups showed a high level of acquisition of practical basic life support skills, with consistent results in most basic maneuvers. The only significant differences were observed in automated external defibrillator management (OR = 3.19; *p* = 0.06), with better performance among students trained by healthcare professionals. The model based on nonhealthcare teachers was associated with improved outcomes, was replicable, and has great potential for expanding basic life support learning in nonclinical university settings. The results are limited by the cross-sectional design and the immediate post-training assessment.

**Discussion:**

Basic life support training for non-healthcare university teachers appears to be a valid and useful approach for disseminating resuscitation knowledge and enhancing society's capacity to respond to life-threatening emergencies. This model can be integrated into cross-disciplinary university training programmemes, strengthening the culture of resuscitation and contributing to a sustainable health and social impact.

## 1 Introduction

Cardiac arrest (CA) is one of the leading causes of death worldwide and represents a major public health problem. It is defined as the sudden, unexpected, and potentially reversible cessation of heart activity and spontaneous breathing, leading to immediate loss of consciousness and, if not treated early, death. Rapid and effective action relies heavily on trained witnesses and the immediate application of basic life support (BLS), the first links in the chain of survival (1, 2).

Early recognition, calling for help, initiating cardiopulmonary resuscitation (CPR), and early defibrillation significantly improve survival after out-of-hospital cardiac arrest (3). Each minute of delay reduces survival chances by approximately 10%, as described by the “Drinker curve” (4). Therefore, formal and regular BLS training of the general population is crucial to reduce mortality.

International organizations, such as the European Resuscitation Council (ERC) and the International Liaison Committee on Resuscitation (ILCOR), together with the American Heart Association (AHA), have established evidence-based CPR guidelines aimed at healthcare professionals and the general public (5, 6). The “Kids Save Lives” initiative, endorsed by the WHO, recommends introducing CPR training in schools from age 12 with annual sessions of at least 2 h, which has been shown to increase bystander CPR rates and out-of-hospital survival (7, 8).

Despite this progress, heterogeneity remains in training delivery, content, and assessment methods. Instructor-led courses generally achieve better outcomes, particularly in psychomotor skills (9), and skills deteriorate after 3–6 months without refresher sessions, highlighting the importance of ongoing practice (10). Traditionally, BLS training has been limited to healthcare professionals, which constrains its reach. Involving teachers as trained instructors has shown promise; secondary school teachers can acquire and transmit CPR skills effectively after structured training (11).

The Cervantes Model demonstrated that non-healthcare teachers can train students with competence comparable to that of healthcare instructors (11). Extending this approach to higher education, the Lives to Give Life project hypothesizes that trained non-healthcare university professors can achieve similar learning outcomes using a cascade model to multiply the educational impact and integrate BLS into university curricula, promoting prevention, social responsibility, and solidarity.

Despite the growing body of evidence supporting cascade training models in basic life support (BLS), the number of studies that systematically compare the ability of non-healthcare instructors to disseminate these skills to the general population remains limited. Moreover, controversy exists within the scientific literature, with some studies considering healthcare professionals as the gold standard for training the general population, although reported outcomes are variable (12).

The aim of this study was to evaluate the benefits and social impact of this innovative BLS teaching model delivered by trained non-healthcare university teachers compared to traditional healthcare-led training. We analyzed the acquisition of theoretical and practical skills by non-healthcare students at the University of Granada (Spain), the fidelity of teachers in reproducing methodologies and skills, and the feasibility of this model as a sustainable strategy to expand BLS knowledge and strengthen a culture of resuscitation in the university community and society.

## 2 Material and methods

### 2.1 Study design

An observational, analytical, and comparative cross-sectional study was conducted as part of the *Lives to Give Life* project at the University of Granada (Spain). The methodological design was based on the recommendations of the European Resuscitation Council (20) and was structured to ensure pedagogical equivalence between the two groups. The aim was to verify whether non-healthcare teachers could acquire the necessary skills to competently teach BLS training and achieve learning outcomes comparable to those obtained from healthcare instructors.

### 2.2 Participants

The sample consisted of 333 university students from different degree programmemes at the University of Granada (Spain). Of these, 121 received training from healthcare professionals and 212 were instructed by previously trained nonhealthcare university teachers. The participants came from degrees representing three major academic areas: Science, Technology and Engineering, and Social and Legal Sciences. The selection was made using nonprobabilistic convenience sampling, including students from the first to fifth years of both sexes and aged between 18 and 30. The inclusion criteria were as follows: being enrolled in a non-health-related university degree programmeme and voluntarily agreeing to participate. The exclusion criteria were students who did not complete the training session or whose evaluation could not be carried out properly. Participation was completely voluntary and anonymous, after reading and signing 130 the informed consent form.

### 2.3 Procedure

The study was conducted in two consecutive phases.

- Phase 1: training of nonhealthcare teachers. A group of university professors received intensive theoretical and practical training in BLS techniques based on the updated ERC guidelines (2021).
- Phase 2: educational intervention with students. The trained teachers then replicated the same BLS programmeme with their students, following a standardized methodology that ensured consistency between the two groups. The control group was instructed by healthcare professionals using the same structure, materials, and evaluation criteria.

For this purpose, a Basic Life Support (BLS) training programmeme was designed, based on the international recommendations established by the European Resuscitation Council (ERC), including training in automated external defibrillation (AED). The instructional units of this training programmeme were structured as follows:

1. Conceptual training: cardiopulmonary arrest, cardiopulmonary and cerebral resuscitation, chain of survival, BLS, CPA epidemiology, bioethical principles applied to life support practice, and the legal framework.
2. Practical training: CPR workshop; airway obstruction management workshop; automated external defibrillation workshop; action sequences according to the Integrated BLS Workshop model.

Each training session was limited to a maximum of 25 participants per course and was delivered by a minimum of two instructors. The course duration was approximately 4 h but could be extended depending on participants' needs, particularly if additional time was required for practical training.

At the beginning of the conceptual training, during the first part of the course, a brief theoretical introduction session (microlearning) was delivered, aimed at facilitating the transmission and acquisition of the most fundamental concepts before applying them in practical simulation.

Following this, practical training commenced based on the demonstrative method. Instructors applied this methodology to the different techniques that comprise BLS, namely:

1. CPR workshop
2. Airway obstruction management
3. Automated external defibrillation workshop

Finally, each of the previously practiced maneuvers was performed in an integrated manner according to the Integrated BLS Workshop model.

After addressing all pertinent questions, participants were allowed to practice with the simulation materials, during which they could also clarify any additional doubts. Once the instructors considered that the participants had acquired and integrated the necessary knowledge and technical skills, the evaluation phase was initiated.

Following the training of non-healthcare faculty members, we initiated the training of students from non-healthcare degree programmemes. These courses followed the same structure and used the same elements and materials as the courses described for non-healthcare faculty members, assessing the same competencies.

Although the courses contained identical content, they differed according to the type of instructor: one group of students was taught by healthcare personnel (control group), whereas another group (intervention group) was instructed by non-healthcare faculty members who had been trained in the previous phase.

Each course delivered by non-healthcare instructors was continuously supervised by a healthcare professional with experience in cardiopulmonary resuscitation, who was assigned to monitor the training sessions. The role of the supervisor was to observe the instructional process and identify any potentially significant errors in the delivery of basic life support content. In the event that such errors were detected, they would have been reported to the study coordinators and subsequently addressed through clarification and corrective explanation to the students at the end of the session, and this information would have been considered in the statistical analysis. However, no significant errors were observed during any of the supervised sessions. Beyond this structured monitoring and oversight, supervisors did not intervene in the course delivery or influence its progression.

### 2.4 Variables and instruments

Sociodemographic variables (age, sex, degree, academic year, and previous CPR experience) and variables related to practical BLS performance were recorded. The skills assessed included: recognition of CPR and request for help, opening the airway using the head-tilt chin-lift maneuvers, correct position of hands, knees, and elbows, depth and frequency of chest compressions (100–120/min and ≥5 cm), complete chest recoil, and AED use (turning on, placing pads, and following instructions). The assessment was performed through structured direct observation using a standardized checklist based on the ERC (2021) recommendations. Each item was rated correct or incorrect. The observers were external, certified instructors who were not involved in the intervention to avoid assessment bias.

### 2.5 Quality control and reliability

To ensure internal validity and comparability of the results, the following measures were applied: the same methodology, content, and materials were used in both groups. The evaluators were independent. A constant ratio of 1 instructor for every 6 participants was maintained during the sessions. The homogeneity of the teaching was verified through random observation of the sessions by the coordinating team. These actions ensured the reproducibility of the process and consistency in the application of the evaluation criteria.

### 2.6 Statistical analysis

A descriptive and inferential analysis of the recorded variables was performed. Qualitative variables were expressed as absolute frequencies and percentages; quantitative variables were expressed as means and standard deviations. To compare proportions between the groups of healthcare professionals and non-healthcare teachers, Pearson's chi-square test was applied, and in cases with expected frequencies < 5, Fisher's exact test was applied. The Student's *t*-test for independent samples was used for continuous variables. Odds ratios (OR) with 95% confidence intervals (95% CI) were also calculated as a measure of association between type of training and level of performance in BLS. A *p*-value < 0.05 was considered statistically significant. Data analysis was performed using IBM SPSS Statistics^®^ v.26. (IBM Corp., Armonk, NY, USA).

To avoid variability in the data attributable to individual instructors and to ensure comparability across the different training sessions—thereby preventing an overestimation of statistical significance—a multivariate logistic regression analysis was performed. This approach allowed adjustment for potential instructor- and training-related confounders, providing more robust and reliable estimates of associations of interest.

### 2.7 Ethical considerations

The study was conducted following the principles of the Declaration of Helsinki (2013 revision) and European data protection regulations (EU Regulation 2016/679). All participants signed an informed consent form before their inclusion. The study protocol was evaluated and approved by the Ethics Committee of the University of Granada (Spain; code LGL2024-UGR). The information obtained was treated confidentially and anonymously, and the data were used exclusively for research.

## 3 Results

The analysis included 333 university students who completed basic life support (BLS) training as part of the *Lives to Give Life* project. Of these, 121 (36.3%) received instruction from healthcare professionals, while 212 (63.7%) were trained by non-healthcare university teachers who had previously been trained through a theoretical-practical course structured in accordance with the guidelines of the European Resuscitation Council (ERC, 2021). The two groups were comparable in age, sex, and academic characteristics, ensuring the homogeneity of the sample and validity of the comparisons.

### 3.1 Sociodemographic and educational characteristics

The descriptive variables are presented in Table 1. The mean age was 20.8 ± 1.9 years in the group formed by healthcare workers and 20.5 ± 2.0 years in the group taught by university professors. Females predominated in both groups, with a slight difference in favor of the healthcare group (76 vs. 65%). Most participants were in their second to fourth year of undergraduate studies. Twenty-three percent of all students had received some prior training in CPR, while less than 10% reported having witnessed or participated in an actual cardiac arrest. These data reflect a low level of initial experience that was comparable between groups.

**Table 1:** General characteristics of the sample.

**Variable**	**Category**	**Healthcare professionals (*n* = 121)**	**Teachers (*n* = 212)**
Age (years)	Mean ± SD	20.8 ± 1.9	20.5 ± 2.0
Gender	Female/male	76%/24%	65%/35%
Academic year	1st−2nd/3rd−4th/≥5th	32%/60%/8%	44%/53%/3%
Previous CPR course	Yes/no	19%/81%	26%/74%
Previous CPR training	Yes/no	6%/94%	7%/93%

Distribution of participants according to age, gender, academic year, previous CPR course, and previous CPR training.

Both groups started from equivalent conditions regarding age, academic level, and prior exposure to CPR, which reinforces the comparability of the results obtained.

### 3.2 Comparison of practical maneuvers between groups

Differences in the acquisition of CPR skills were evaluated between two groups of students: those trained by healthcare professionals (Type 1) and those trained by university professors (Type 2). Odds ratios (OR) with corresponding 95% confidence intervals (95% CI) were estimated to compare performance across CPR maneuvers, AED use, and resuscitation assistance by performing a bivariate analysis using adjusted odds ratios (Haldane–Anscombe correction), when appropriate (Table 2).

**Table 2:** Comparison of practical maneuvers between the group trained by healthcare professionals and the group trained by university professors using bivariate analysis with adjusted odds ratios (Haldane–Anscombe correction) when appropriate.

**Variable**	**Type 1 no (%)**	**Type 1 yes (%)**	**Type 2 no (%)**	**Type 2 yes (%)**	**OR**	**95% CI inf**	**95% CI sup**	***p*-value**
Previous CPR training	98 (81.00)	23 (19.00)	158 (74.53)	54 (25.47)	1.456	0.841	2.522	0.178
Previous PCR care	114 (94.21)	7 (5.79)	198 (93.33)	14 (6.67)	1.152	0.452	2.936	0.768
Assessing consciousness	5 (4.12)	116 (95.88)	11 (5.22)	201 (94.78)	0.788	0.267	2.323	0.665
Calling for help	9 (7.41)	112 (92.59)	9 (4.24)	203 (95.76)	1.813	0.699	4.697	0.215
Placing the patient face up	4 (3.33)	117 (96.67)	2 (0.94)	210 (99.06)	3.590	0.648	19.895	0.195
Head-tilt chin-lift maneuver	9 (7.41)	112 (92.59)	6 (2.83)	206 (97.17)	2.759	0.957	7.950	0.051
See-feel-hear maneuver	3 (2.46)	118 (97.54)	8 (3.77)	204 (96.23)	0.648	0.169	2.491	0.752
Calling 911	7 (5.79)	114 (94.21)	7 (3.30)	205 (96.70)	1.798	0.615	5.255	0.277
Requests the AED	15 (12.42)	106 (87.58)	9 (4.24)	203 (95.76)	**3.192**	**1.352**	**7.536**	**0.006**
Correct position of the hands	4 (3.33)	117 (96.67)	10 (4.66)	202 (95.34)	0.691	0.212	2.251	0.777
Correct position next to the patient	1 (0.83)	120 (99.17)	5 (2.37)	207 (97.63)	0.345	0.040	2.988	0.423
Extended elbows	2 (1.68)	119 (98.32)	11 (5.22)	201 (94.78)	0.307	0.067	1.409	0.145
Compress using the trunk	5 (4.12)	116 (95.88)	3 (1.42)	209 (98.58)	3.003	0.705	12.792	0.145
Compress to a depth of 5 cm	9 (7.41)	112 (92.59)	6 (2.83)	206 (97.17)	2.759	0.957	7.950	0.051
100–120 compression per minute	4 (3.33)	117 (96.67)	7 (3.30)	205 (96.70)	1.001	0.287	3.492	1.000
Chest recoil after compression	3 (2.46)	118 (97.54)	3 (1.42)	209 (98.58)	1.771	0.352	8.915	0.672
Turn on the AED	4 (3.33)	117 (96.67)	0 (0.00)	212 (100.00)	**Inf**	**Nan**	**Inf**	**0.017**
Apply the patches correctly	5 (4.12)	116 (95.88)	8 (3.77)	204 (96.23)	1.099	0.351	3.438	0.871
Follow the AED's instructions	2 (1.68)	119 (98.32)	3 (1.42)	209 (98.58)	1.171	0.193	7.107	1.000
Coordinates compression with AED orders	2 (1.68)	119 (98.32)	4 (1.89)	208 (98.11)	0.874	0.158	4.843	1.000

Results are expressed as adjusted odds ratios (OR) with 95% confidence intervals and *p*-values. Values highlighted in bold indicate statistically significant results.

To account for instructor-related variability, ensure comparability across training sessions, and avoid overestimation of statistical significance, a penalized logistic regression using Firth's method was performed (Table 3). This approach was selected to reduce small-sample bias and obtain stable and reliable estimates in situations where some outcomes presented sparse data or complete or quasi-complete separation between groups, conditions under which conventional maximum likelihood logistic regression may yield infinite or unstable estimates. The method allowed the inclusion of variables affected by separation and provided robust estimates of ORs and 95% CIs. The model included 22 variables related to CPR maneuvers, AED use, and assistance during resuscitation, with student type coded as 0 = university student and 1 = healthcare-related student.

**Table 3:** Penalized firth logistic regression analysis of the study variables.

**Variable**	**OR (IC 95%)**	***p*-value**
Previous CPR training	1.350 (0.762–2.448)	0.307
Previous PCR care	0.923 (0.355–2.605)	0.873
Assessing consciousness	0.771 (0.223–2.296)	0.650
Calling for help	1.303 (0.429–3.842)	0.633
Placing the patient face up	1.185 (0.141–9.082)	0.867
Head-tilt chin-lift maneuver	6.417 (1.463–38.556)	0.013
See-feel-hear maneuver	0.316 (0.040–1.616)	0.176
Calling 911	0.596 (0.074–3.627)	0.582
Requests the AED	4.087 (1.412–13.304)	0.009
Correct position of the hands	0.702 (0.192–2.179)	0.552
Correct position next to the patient	0.382 (0.023–2.906)	0.379
Extended elbows	0.223 (0.020–1.112)	0.070
Compress using the trunk	**6.270 (1.140–64.608)**	**0.034**
Compress to a depth of 5 cm	1.757 (0.551–5.600)	0.333
100–120 compression per minute	0.648 (0.118–2.747)	0.567
Chest recoil after compression	1.870 (0.179–30.690)	0.599
Turn on the AED	5.324.8 (8.02–4,421.910)	0.001
Apply the patches correctly	0.576 (0.133–2.096)	0.419
Follow the AED's instructions	0.060 (0.000–1.732)	0.116
Coordinates compression with AED orders	0.043 (0.000–0.841)	0.036

Multivariate analysis assessing the association between instructor profile and performance in basic life support maneuvers. Penalized Firth logistic regression was applied to reduce small-sample bias and handle complete or quasi-complete separation. Results are presented as odds ratios (OR) with 95% confidence intervals. Values highlighted in bold indicate statistically significant results.

Overall, students trained by healthcare professionals showed a higher likelihood of correctly performing several critical CPR and AED-related actions. In particular, requesting an AED was significantly more frequent among students trained by healthcare professionals, who were more likely to request an AED than those trained by university professors (OR = 4.09; 95% CI 1.41–13.30; *p* = 0.009). Similarly, turning on the AED showed an extremely high OR (*p* = 0.001), reflecting that almost all healthcare-trained students correctly performed this maneuver, resulting in complete separation between groups. In some items—particularly AED activation—complete or quasi-complete separation was observed; therefore, the very large odds ratios should be interpreted primarily as evidence of a strong directional association rather than as precise estimates of effect magnitude.

Significant differences were also observed for other key CPR maneuvers. Students trained by healthcare professionals were more likely to correctly perform the head-tilt chin-lift maneuver (OR = 6.42; 95% CI 1.46–38.56; *p* = 0.013) and perform chest compressions using the trunk (OR = 6.27; 95% CI 1.14–64.61; *p* = 0.034). These findings suggest superior performance in more technically demanding maneuvers among students trained by healthcare professionals.

In contrast, some isolated skills showed better performance among university students. Specifically, coordination of chest compression with AED voice prompts was more frequent in this group (OR = 0.043; 95% CI 0.000–0.841; *p* = 0.036). For other variables—including previous CPR training, previous CPR care, calling for help, placing the patient face up, performing chest compressions, and calling emergency services—no statistically significant differences were observed between groups (*p* > 0.05).

Globally, both groups demonstrated high levels of competence, with performance rates exceeding 90% for most basic CPR maneuvers. The main differences were concentrated in AED-related actions, particularly AED request and activation, which may be explained by the healthcare instructors' greater prior familiarity with this device.

**Figure 1:**
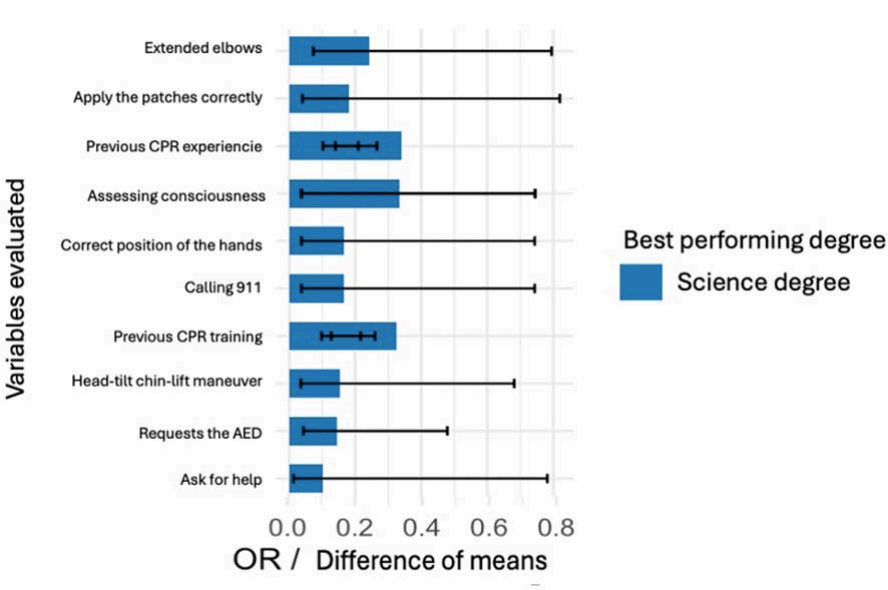
Performance in CPR according to degree.

The overall model demonstrated good explanatory capacity. The likelihood ratio test confirmed that the set of variables included in the model was statistically significant (χ^2^ = 38.27, 22 df, *p* = 0.017), and the global Wald test was also significant (χ^2^ = 47.64, 22 df, *p* = 0.001), indicating that the included variables discriminated between students trained by healthcare professionals and those trained by university professors. The total sample size was *n* = 333 students. Figure 1 presents the forest plot of the odds ratios.

Although most basic life support (BLS) skills were acquired at similar levels in both training groups, statistically significant differences were concentrated specifically on actions related to the use of the automated external defibrillator (AED). Students trained by healthcare instructors were particularly more likely to request an AED (OR = 4.087; 95% CI 1.412–13.304; *p* = 0.009) and correctly turn on the device (highly significant association; *p* = 0.001). In contrast, no significant differences were observed between groups in most core BLS maneuvers, including initial assessment, calling for help, quality of chest compressions, and basic sequence of action.

### 3.3 Analysis by sex

The analysis stratified by sex revealed that, in general terms, performance was similar between men and women. However, two statistically significant differences were detected. Thus, the head-tilt chin-lift maneuver was performed correctly by a higher proportion of men (OR = 3.521; 95% CI: 1.235–10.763; *p* = 0.020). Chest recoil after compression also showed better results in men (OR = 11.515; 95% CI: 1.826–222.146; *p* = 0.027). No significant differences were found in the other skills (compressions, request the AED, calling for help, and assessing consciousness).

### 3.4 Analysis by age

The age of the students was compared with different dichotomous variables related to CPR course skills. No significant age differences were observed in most of the course variables: previous CPR course, previous CPR care, request the AED, head tilt chin lift maneuver, calling for help, etc., indicating that age does not significantly influence the acquisition of these skills or behavior during training. The variable “correct position of hands” showed a *p*-value of 0.005, indicating that there was a significant age difference between the groups that performed the chest compression maneuver correctly, suggesting that, for this specific skill, age could have some effect, although the analysis is exploratory and does not establish causality. The mean age of those who performed the maneuver correctly was higher (20.7 years) than that of those who did not perform it correctly (19.3). Thus, older students tended to perform the cardiac massage maneuver better. Overall, the differences observed are minimal, and most of the high *p*-values confirm that there is no statistically significant association.

### 3.5 Analysis according to university degree

**Figure 2:**
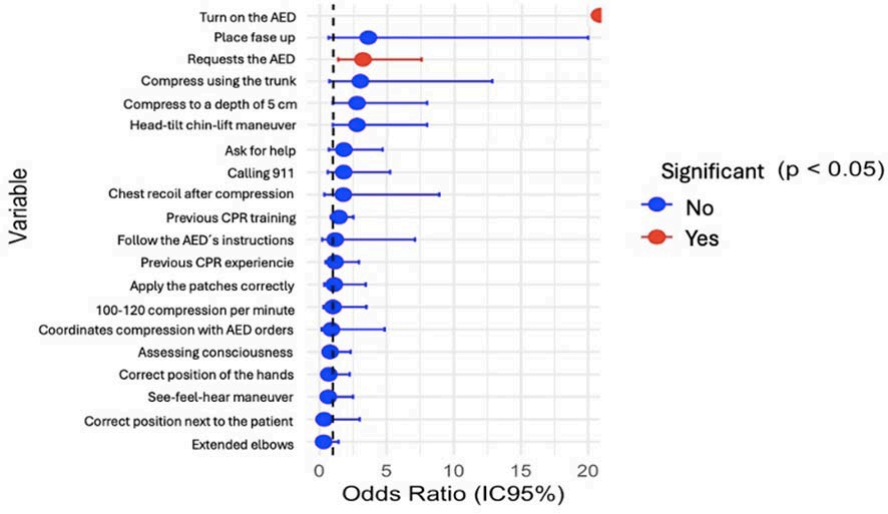
Forest plot of OR by dichotomous variable.

A significant relationship was observed between the degree studied and the level of performance in the practical maneuvers. Variables calling 911, previous CPR care, calling for help, assessing consciousness, previous CPR course, request the AED, head tilt chin lift maneuver, and extended elbows showed significant differences according to degree (*p* < 0.05). In all these variables, Science Degree students (1) performed better, with a higher probability of correctly performing the maneuvers or achieving optimal theoretical results. The Student Type variable also showed a significant difference (ANOVA *p* = 0.005), reinforcing that prior training and degree influence performance. Other variables evaluated did not show significant differences, indicating that the course performs well across all degree programmes, with better outcomes observed in Science. Figure 2 shows CPR performance according to degree.

## 4 Discussion

### 4.1 Main findings

This study provides evidence on the feasibility and potential benefits of the Lives to Give Life educational model, in which non-healthcare university teachers, after receiving structured training in basic life support (BLS), provide practical teaching to their students. The results show that students trained by these teachers achieved levels of competence comparable to those trained directly by healthcare professionals, confirming the validity of the cascade approach as a tool for educational and social expansion.

Overall, the rates of correct execution of basic maneuvers exceeded 90% in both groups, reflecting a high level of practical learning. Students trained by healthcare professionals demonstrated superior performance in complex CPR maneuvers and AED use, whereas university students maintained comparable basic competencies and, in some cases, showed better performance in specific isolated maneuvers. Firth's penalized logistic regression was used to address complete separation, providing reliable estimates of odds ratios and 95% confidence intervals. These findings suggest that, under standardized conditions, non-healthcare instructors can successfully transmit the essential components of BLS.

### 4.2 Comparison between healthcare and non-healthcare instructors

Despite the overall similarity in performance, significant differences were observed in the use and activation of the automated external defibrillator (AED), with better results among students trained by healthcare professionals. This finding is consistent with previous studies by Ali et al. (13) and Díaz-Castellanos and Cárdenas (14), which highlight that instrumental skills requiring technical precision and familiarity with clinical equipment are more strongly influenced by professional practice.

The greater familiarity of healthcare instructors with AED operating sequences, their routine interaction with medical devices, and their confidence in rapid decision-making may explain this advantage. However, the absence of significant differences in other key maneuvers—such as assessment of consciousness, airway opening, chest compressions, and calling for help—indicates that instruction provided by non-healthcare teachers is associated with strong learning outcomes in the core elements of BLS.

A key finding of this study is that the differences between training models were not evenly distributed across all the assessed skills but were concentrated in critical actions related to the use of the AED. Although overall performance in basic BLS maneuvers was high and comparable between groups, students trained by healthcare professionals showed a higher probability of requesting and correctly activating the AED, indicating a focal gap in a central link of the chain of survival. This should not be interpreted as an overall limitation of the cascade teaching model but rather as a priority target for refinement when implementing such models in non-clinical environments. Early defibrillation is one of the most important determinants of survival in cardiac arrest due to shockable rhythms, and correct AED activation requires not only technical skills but also cognitive processes such as rapid decision-making, prioritization of interventions, and operational familiarity with the device. The observed pattern suggests that, while the cascade model is effective for acquiring most BLS skills, the AED component may benefit from complementary strategies, such as focused reinforcement of the activation sequence through deliberate practice with immediate feedback, integration of AED use into full resuscitation scenarios, and, where appropriate, hybrid instructional models in which the AED module is co-delivered or periodically audited by healthcare professionals, while preserving the scalability of the overall programme.

These findings are consistent with those reported by Pérez-Bailón et al. (11) in the Cervantes Model©, where secondary school teachers achieved teaching competence comparable to that of healthcare professionals following standardized training (3).

### 4.3 Educational model and public health implications

The present results reinforce the potential of cascade training models, in which knowledge acquired by a group of trained instructors is exponentially disseminated throughout the population. From a public health perspective, this approach addresses the need to expand cardiopulmonary resuscitation (CPR) training coverage without relying exclusively on healthcare resources.

This strategy aligns with international recommendations from the European Resuscitation Council (ERC), the World Health Organization (WHO), and the Kids Save Lives initiative, which promote the training of teachers as multipliers of CPR knowledge across educational levels, from schools to universities (4–6).

The university setting offers an optimal environment for implementing this model, allowing BLS to be integrated as a cross-cutting competence that strengthens social responsibility and prepares students to respond to life-threatening emergencies.

### 4.4 Role of pedagogy and instructor characteristics

Recent research has shown that educational programmes based on simulation, guided practice, and immediate feedback, even when conducted by non-healthcare trainers, significantly improve the acquisition of psychomotor skills (7, 8). In addition, the instructor's motivation and teaching skills play a decisive role in learning retention, often comparable to or even greater than the influence of their professional profile (9).

Our results support this perspective: non-healthcare university teachers successfully conveyed the fundamentals of BLS, suggesting that teaching competence and adherence to standardized protocol are more decisive than the trainer's profession. This finding is consistent with the study by Fujiwara et al. (15), in which peer-led training achieved similar results to that led by healthcare professionals in teaching BLS. It also coincides with the review by Ng et al. (3), which highlights the effectiveness of alternative training modalities (self-learning, simulation, and peer teaching) as long as adequate structure and supervision are maintained.

### 4.5 Influence of academic background and sex

A slight superiority observed among students with science degrees may be related to greater familiarity with biological or physiological content, as well as a more analytical approach to problem solving, factors previously identified by Mollo et al. (16) in their review of teacher-led CPR programmes. These characteristics may facilitate the assimilation of systematic protocols, such as those of BLS. Nevertheless, the consistent performance of students from other academic areas reinforces the idea that the acquisition of BLS skills does not depend on the area of study but on the quality of the educational process.

Regarding sex-related differences, performance in chest recoil during compression was slightly higher among male students. This difference appears to be related to biomechanical factors, such as physical strength or body composition, which may facilitate the correct execution of this particular skill rather than indicating disparities in learning. No notable differences were observed in other competencies, including chest compressions, AED operation, calling for help, or assessment of consciousness, reinforcing the idea that overall learning outcomes were not affected by sex.

### 4.6 Pedagogical and social impact

From a pedagogical perspective, this work makes a significant contribution by demonstrating that non-health university professors can act as multipliers of health knowledge within higher education institutions. The systematic implementation of this model could transform universities into proactive public health environments, where every student, regardless of their degree, acquires potentially life-saving skills. This idea is aligned with the framework of university social responsibility proposed by UNESCO and the Sustainable Development Goals (SDGs), especially SDG 3 (health and wellbeing) and SDG 4 (quality education) (17).

### 4.7 Limitations

This study has limitations that should be considered. The observational design and immediate post-training assessment of competencies limit the interpretation of the findings to short-term skill acquisition and performance under evaluation conditions. Consequently, no conclusions can be drawn regarding long-term skill retention or sustained competence, which are known to decline over time without regular practice, as documented by Bylow et al. (9) and Boet et al. (18), who reported a significant deterioration in CPR skills after 3–6 months.

Furthermore, the assessment of students across the different courses was observational in nature. Although efforts were made to standardize the criteria required to pass each item, observer bias cannot be completely ruled out, particularly given the technical nature of the assessed maneuvers. In addition, simulators equipped with real-time feedback or capable of objectively quantifying variables such as compression depth and rate were not used. As a result, the assessment of these parameters relied on direct observation, which may have limited the precision and objectivity of the evaluation of specific psychomotor skills, despite the use of standardized assessment criteria.

Although the cascade educational model proved effective for the overall acquisition of BLS competencies among non-health university students, the differences observed in critical AED-related actions suggest that this specific component may require additional reinforcement. While global performance was high, variability in AED activation sequences indicates that certain instrumental steps—such as requesting the device, turning it on, correctly placing the pads, and following voice prompts—may benefit from more targeted instructional strategies. In addition, because statistical separation was observed in some AED-related items, extremely large odds ratios should be interpreted with caution. Since separation can inflate point estimates, these values reflect a focal difference in performance—particularly in AED activation—rather than a literal measure of clinical magnitude. This interpretation further supports the need for specific educational strategies aimed at strengthening the AED module within cascade-based programmes.

Although the sample size was sufficient to detect relevant differences in the main variables, it may have limited the identification of more subtle variations. Subjective indicators such as self-confidence, perceived self-efficacy, or participant satisfaction were not analyzed, despite their potential value in understanding the emotional and attitudinal impact of the programme (19).

The use of convenience sampling may have introduced selection bias, including a potential overrepresentation of highly motivated students. This approach was necessary due to feasibility constraints within the academic setting, but it may limit the generalizability of the findings. Future studies should consider randomized or multicenter sampling strategies to improve external validity.

Another limitation is the scarcity of directly comparable experiences in the scientific literature. Previous studies have primarily focused on training medical students as BLS instructors or on evaluating trained teachers in primary and secondary education settings. Consequently, although our findings are consistent with these experiences, an exact comparison cannot be established. Nevertheless, this study opens a new line of research with potential implications for scaling BLS education in the general population.

From an implementation perspective, future iterations of the programme could incorporate complementary strategies specifically aimed at strengthening AED performance while preserving the scalability of the cascade structure. These may include brief, repeated “AED booster” micro-sessions focused on the activation sequence (request–power on–pad placement–follow prompts), scenario-based training integrating full resuscitation cases to consolidate decision-making and coordination with voice instructions, and the use of cognitive checklists or visual aids to reduce omissions during early learning phases. In addition, a hybrid instructor model could be considered, in which the cascade approach is maintained for core BLS competencies while AED modules are co-delivered or periodically supervised by healthcare professionals, particularly during initial implementations or as part of quality audits. Periodic refresher sessions may also be advisable, prioritizing AED skills given their relative variability and the known decline of CPR-related competencies over time.

Finally, while BLS and CPR education inherently requires a high level of commitment from instructors, the project received an overwhelmingly positive response, with exceptional levels of engagement and willingness to participate.

### 4.8 Future directions

In light of these results, future research should expand the model to include longitudinal evaluations, periodic refresher training, and the incorporation of educational technologies (simulators, apps, and adaptive learning) to improve skill retention. A hybrid model could also be explored, in which university teachers lead theoretical–practical instruction while healthcare professionals supervise sessions focused on AED use and coordination with emergency services.

Overall, the obtained results consolidate the value of the Lives to Give Life model as an innovative and reproducible proposal for basic life support training within the university environment. By demonstrating that the competence of non-healthcare trainers can match that of healthcare professionals in teaching critical maneuvers, this study provides a solid empirical basis for the expansion of BLS in non-clinical settings, contributing to a society that is better prepared to respond to life-threatening emergencies.

## 5 Conclusions

Basic life support (BLS) training provided by previously trained nonhealthcare university teachers appears to be associated with learning outcomes comparable to those achieved with training provided by healthcare professionals. With the exception of the technical skill related to the use and activation of the automated external defibrillator (AED), the results suggest that essential cardiopulmonary resuscitation maneuvers can be taught with similar precision, safety, and pedagogical quality. The Lives to Give Life educational model seems to demonstrate its validity and potential applicability as an innovative and sustainable training strategy. This cascade approach could expand BLS teaching coverage within the university environment, transforming nonhealthcare teachers into multipliers of knowledge in life-threatening emergencies. Its implementation may promote health literacy within the academic community as well as the consolidation of a university culture of prevention, responsibility, and social commitment.

Notably, at present, these results are primarily applicable to higher education institutions in Europe; a future line of research would be to evaluate the model's feasibility and effectiveness across a broader range of higher education settings globally.

Integrating BLS training as a cross-cutting skill in university curricula may contribute to international health and education objectives promoted by the WHO, ERC, and UNESCO, aligning with SDG 3 (Good Health and Wellbeing) and SDG 4 (Quality Education), by facilitating short-term acquisition of essential life-saving skills in a broad student population. However, the present findings are limited to immediate post-training outcomes. Future research should therefore focus on evaluating long-term learning retention, the effectiveness of periodic reinforcement strategies, and the translation of these short-term competencies into real-world performance and willingness to act in the event of a cardiac arrest. In this context, the incorporation of educational technologies, interactive simulators, and monitoring platforms may help support skill maintenance and improve the sustainability of training effects over time.

Overall, this work provides preliminary scientific evidence and practical insights for incorporating BLS into non-healthcare university education, positioning the Lives to Give Life model as a potentially transformative, replicable, and high-impact approach for preparing citizens to act in life-threatening emergencies.

## Data Availability

The original contributions presented in the study are included in the article/Supplementary material, further inquiries can be directed to the corresponding author.
