# Supplementary material for: Comparative evaluation of a basic life support educational model in non-health university students: the “Lives to Give Life” project and its social and health impact
Source: Front Med (Lausanne). 2026 Mar 10;13:1765154. doi: 10.3389/fmed.2026.1765154 (PMC13008744; doi:10.3389/fmed.2026.1765154)
Supplement: Supplementary file 1 [file Data_Sheet_1.pdf]

Supplementary Material

Table S1. Frequencies and percentages of students belonging to the Control Group and the Intervention Group.

	FREQUENCY	PERCENTAGE (%)
**Control Group**	121	36.3
**Intervention Group**	212	63.66
**Total**	333	100

Table S2. Age distribution of students belonging to the Control Group and the Intervention Group.

	AGE
**Control group**	20.8 ± 1.9 years
**Intervention group**	20.5 ± 2.0 years

Table S3. Distribution of the variable Gender in both groups.

	FEMALE	MALE	TOTAL
**Control group**	92 (76%)	29 (24%)	121 (100%)
**Intervention group**	138 (65%)	74 (35%)	212 (100%)

Table S4. Distribution of the variable Nationality according to the Control Group and the Intervention Group.

NATIONALITY	CONTROL GROUP (%)	INTERVENTION GROUP (%)
**Spanish**	93.5	99
**Spanish-German**	0.8	0
**German**	0	0.5
**Argentina**	0.8	0
**Bolivian**	0.8	0
**Colombian**	0.8	0
**Ecuadorian**	0	0.5
**Italian**	0	0.5
**Moroccan**	1.7	0
**Portuguese**	0.8	0

Table S5. Distribution of the variable Qualification according to the Control Group and the Intervention Group.

DEGREE	CONTROL GROUP (%)	INTERVENTION GROUP (%)
**Biology**	32.3	13.1
**Biochemistry**	5	2.4
**Biotechnology**	11.6	6.6
**Environmental sciences**	7.4	3.8
**Sports science**	0	18.4
**Law**	0.8	0
**Early childhood education**	0.8	16
**Primary education. French studies**	0	1.4
**Industrial electronic engineering**	5.8	5.2
**Statistics**	2.5	0.5
**Pharmacy**	0.8%	0%
**Physics**	6.6	15.6
**Geology**	0.8	0.5
**Chemical engineering**	8.2	1.9
**Mathematics**	2.5	2.8
**Mathematics. Physics**	5	1.4
**Optics**	2.5	4.7
**Education**	0	0.5
**Psychology**	0.8	0
**Chemistry**	6.6	5.2
**Total**	100	100

Table S6. Distribution of the variable Academic year, according to the Control Group and the Intervention Group.

	CONTROL GROUP (%)	INTERVENTION GROUP (%)
**1st - 2nd**	32	44
**3rd - 4th**	60	53
**5th or higher**	8	3
**Total**	100	100

Table S7. Distribution of the variable Previous CPR course, according to the Control Group and the Intervention Group.

PREVIOUS COURSE CPR	CONTROL GROUP (%)	INTERVENTION GROUP (%)
**Yes**	19	25
**No**	81	75
**Total**	100	100

Table S8. Distribution of the variable Previous CPR care, according to the Control Group and the Intervention Group.

PREVIOUS CPR TRAINING	CONTROL GROUP (%)	INTERVENTION GROUP (%)
**Yes**	6	7
**No**	94	93
**Total**	100	100

Table S9. Percentages of each of the study variables, according to whether they are performed correctly or not. in the Control Group and in the Intervention Group.

VARIABLE	CONTROL GROUP		INTERVENTION GROUP	
	Performed (%)	Not Performed (%)	Performed (%)	Does not Perform (%)
Assessing consciousness	96	4	95	5
Ask for help	93	7	96	4
Place face up	97	3	99	1
Head-tilt chin-lift maneuver	93	7	97	3
See-feel-hear maneuver	98	2	96	4
Calling 911	94	6	97	3
Requests the AED	98	2	96	4
Correct position of the hands	97	3	95	5
Correct position next to the patient	99	1	98	2
Extended elbows	98	2	95	5
Compress using the trunk	96	4	99	1
Compress to a depth of 5 cm	93	7	97	3
100-120 compression per minute	97	3	97	3
Chest recoil after compression	98	2	99	1
Turn on the AED	97	3	100	0
Apply the patches correctly	96	4	96	4
Follow the AED´s instructions	98	2	99	1
Coordinates compression with AED orders	98	2	98	2

Table S10. Comparison of skill acquisition between the control group and the intervention group. Variables showing statistically significant differences are highlighted in red.

VARIABLE	OR	CI 95% INF	CI 95% SUP	P value
Assessing consciousness	0.788	0.267	2.323	0.665
Ask for help	1.813	0.699	4.697	0.215
Place face up	3.590	0.648	19.895	0.195
Head-tilt chin-lift maneuver	2.759	0.957	7.950	0.051
See-feel-hear maneuver	0.648	0.169	2.491	0.752
Calling 911	1.798	0.615	5.255	0.277
Requests the AED	**3.192**	**1.352**	**7.536**	**0.006**
Correct position of the hands	0.691	0.212	2.251	0.777
Correct position next to the patient	0.345	0.040	2.988	0.423
Extended elbows	0.307	0.067	1.409	0.145
Compress using the trunk	3.003	0.705	12.792	0.145
Compress to a depth of 5 cm	2.759	0.957	7.950	0.051
100-120 compression per minute	1.001	0.287	3.492	1.000
Chest recoil after compression	1.771	0.352	8.915	0.672
Turn on the AED	**Inf**	**NaN**	**Inf**	**0.017**
Apply the patches correctly	1.099	0.351	3.438	0.871
Follow the AED´s instructions	1.171	0.193	7.107	1.000
Coordinates compression with AED orders	0.874	0.158	4.843	1.000

Table S11. Comparison of skill acquisition according to student gender. Variables showing statistically significant differences are highlighted in red.

VARIABLE	OR	CI 95% INF	CI 95% SUP	P value
Assessing consciousness	0.583	0.723	0.198	2.135
Ask for help	0.085	2.316	0.878	6.109
Place face up	0.911	1.103	0.152	5.745
Head-tilt chin-lift maneuver	0.020	3.521	1.235	10.763
See-feel-hear maneuver	0.774	0.821	0.177	2.903
Calling 911	0.712	1.235	0.371	3.669
Requests the AED	0.818	1.109	0.437	2.613
Correct position of the hands	0.826	0.876	0.236	2.688
Correct position next to the patient	0.911	1.103	0.152	5.745
Extended elbows	0.568	1.395	0.413	4.289
Compress using the trunk	0.701	0.729	0.106	3.225
Compress to a depth of 5 cm	0.344	0.537	0.120	1.735
100-120 compression per minute	0.709	1.269	0.326	4.299
Chest recoil after compression	0.027	11.515	1.826	222.146
Turn on the AED	0.788	0.731	0.036	5.789
Apply the patches correctly	0.971	0.978	0.260	3.080
Follow the AED´s instructions	0.672	1.477	0.192	9.043
Coordinates compression with AED orders	0.911	1.103	0.152	5.745

Table S12. Interpretation of variables showing statistically significant differences after comparing them by gender.

VARIABLE	P_VALUE	OR / MEAN DIFFERENCE	CI 95%	GENDER WITH BETTER PERFORMANCE
Head-tilt chin-lift maneuver	0.020	3.521	1.235 - 10.763	Male
Chest recoil after compression	0.027	11.515	1.826 - 222.146	Male

Table S13. Comparison of skill acquisition according to student age. Variables showing statistically significant differences are highlighted in red.

VARIABLE	AVERAGE ACQUIRES SKILL	AVERAGE DOES NOT ACQUIRE	DIFFERENCE	P-VALUE
Assessing consciousness	20.6	20.0	1	p = 0.228
Ask for help	20.6	20.1	-	p = 0.286
Place face up	20.6	21.3	0	p = 0.192
Head-tilt chin-lift maneuver	20.6	21.0	0	p = 0.227
See-feel-hear maneuver	20.6	21.2	0	p = 0.156
Calling 911	20.6	20.2	0	p = 0.565
Requests the AED	20.6	20.5	0	p = 0.895
Correct position of the hands	20.7	19.3	-2	p = 0.005
Correct position next to the patient	20.5	20.6	0.5	p = 0.932
Extended elbows	20.4	20.6	1	p = 0.824
Compress using the trunk	20.1	21.6	0	p = 0.492
Compress to a depth of 5 cm	20.5	20.6	0	p = 0.864
100-120 compression per minute	20.5	20.6	0	p = 0.869
Chest recoil after compression	20.3	21.6	-0.5	p = 0.240
Turn on the AED	20.2	21.6	-1	p = 0.329
Apply the patches correctly	20.2	20.6	1	p = 0.582
Follow the AED´s instructions	20.6	21.6	-1	p = 0.090
Coordinates compression with AED orders	20.8	20.6	-0.5	p = 0.510

Table S14. Comparison of skill acquisition according to student qualifications.

VARIABLE	OR	CI 95% INF	CI 95% SUP	P value
Assessing consciousness	0.167	0.037	0.745	0.019
Ask for help	0.059	0.008	0.442	0.006
Place face up	0.200	0.023	1.713	0.142
Head-tilt chin-lift maneuver	0.154	0.035	0.682	0.014
See-feel-hear maneuver	0.309	0.087	1.102	0.070
Calling 911	0.166	0.037	0.744	0.019
Requests the AED	0.143	0.043	0.479	0.002
Correct position of the hands	2037.575	0.000	2.076e28	0.795
Correct position next to the patient	0.272	0.044	1.675	0.160
Extended elbows	0.241	0.073	0.795	0.019
Compress using the trunk	464.444	0.000	2.927e18	0.741
Compress to a depth of 5 cm	0.356	0.107	1.186	0.092
100-120 compression per minute	0.306	0.075	1.243	0.098
Chest recoil after compression	332.294	0.000	2.555e18	0.756
Turn on the AED	272.331	0.000	1.113e21	0.798
Apply the patches correctly	3738.657	0.000	1.087e39	0.843
Follow the AED´s instructions	581.257	0	5.926e23	0.796

Table S15. Interpretation of variables showing statistically significant differences after comparing them according to student degree.

VARIABLE	P VALUE	OR / MEAN DIFFERENCE	CI 95% INF	CI 95% SUP	TITLE BEST PERFORMANCE
**Calling 911**	0.019	0.166	0.037	0.744	Science degree
**Assessing consciousness**	0.019	0.167	0.037	0.745	Science degree
**Requests the AED**	0.002	0.143	0.043	0.479	Science degree
**Head-tilt chin-lift maneuver**	0.014	0.154	0.035	0.682	Science degree
**Asks for help**	0.006	0.059	0.008	0.442	Science degree
**Extended elbows**	0.019	0.241	0.073	0.795	Science degree

LIVES TO GIVE LIFE PROJECT

Competency checklist

- Check consciousness using the shout and shake manoeuvre.
    - Perform the shout and shake manoeuvre.
- Call for help (with the questions: Do you have a mobile phone? Do you know CPR?)
- If they do not respond, open the airway using the head-tilt chin-lift manoeuvre.
    - Place the patient on their back.
    - Hold the patient's forehead with one hand.
    - With the fingers of your other hand, move the patient's jaw upwards and forwards.
- While maintaining the head-tilt chin-lift manoeuvre, check for breathing using the look, listen and feel manoeuvre for a maximum of 10 seconds.
    - Maintain the head-tilt chin-lift manoeuvre, with the airway open.
    - Place your head close to the nose and mouth, looking towards the patient's chest.
    - Perform the manoeuvre for less than 10 seconds.
- If they are not breathing: call for help, activate the emergency system and request an AED.
    - Ask other bystanders for help.
    - Activate the emergency system.
    - Request the AED.
- Locate the point for performing chest compressions.
    - Place your hands on the sternum, on the intermammary line, to perform chest compressions.
- Position yourself correctly to perform chest compressions.
    - Kneel beside the patient.
    - Place one hand on top of the other at the previously indicated point.
    - Keep your elbows extended during compressions.
    - Perform compressions using the weight of your torso, not just your arms.
- Perform continuous, high-quality chest compressions correctly.
    - Press down on the chest at least 5 cm.
    - Maintain a rate of 100-120 compressions per minute.
    - Allow the chest to fully re-expand between compressions.
- Use the automated external defibrillator (AED) correctly (AED)
    - Turn on the device.
    - Place the pads on the spot indicated on the device itself.
    - Follow the device's instructions.
    - Alternate continuous, high-quality chest compressions with the AED's commands.
